# Correlative light and volume electron microscopy (vCLEM): How community participation can advance developing technologies

**DOI:** 10.1111/jmi.13056

**Published:** 2021-09-20

**Authors:** Christopher J. Guerin, Saskia Lippens

**Affiliations:** ^1^ VIB Bio Imaging Core VIB − Ghent University Ghent Belgium

**Keywords:** correlative light and electron microscopy, correlative volume electron microscopy, electron microscopy, volume electron microscopy

## Abstract

Correlative light and electron microscopy is a valuable tool to image samples across resolution scales and link data on structure and function. While studies using this technique have been available since the 1960s, recent developments have enabled applying these workflows to large volumes of cells and tissues. Much of the development in this area has been facilitated through the collaborative efforts of microscopists and commercial companies to bring the methods, hardware and image processing technologies needed into laboratories and core imaging facilities. This is a prime example of how what was once a niche area can be brought into the mainstream of microscopy by the efforts of imaging pioneers who push the boundaries of possibility.

In the 1980s when confocal microscopes became commercially available some electron microscopy (EM) labs invested in the technology. Since electron microscopists generally used light microscopy (LM) mostly to examine semi‐thin sections before taking ultra‐thin ones to examine in the transmission electron microscope (TEM), this must have seemed to many an extravagant purchase. However, the confocal was able to reveal the wonder of cell structure in three dimensions (3D), and because of its ability to image at resolutions approaching the Abbe LM limits it also resolved details previously obscured by light scattering from out of focus image planes. This reduced the resolution gap between the two microscopy technologies and thereby made it easier to target EM studies to specific areas. Through newly improved immunohistochemical techniques, as well as better fluorescent probes, electron microscopists were also able to focus their TEM studies to areas where proteins of interest were located. This was in many ways the beginning of modern 3D correlative light and electron microscopy (CLEM). Unfortunately true CLEM where microscopists could use the same probe in the LM and EM was still years away. While confocal microscopy was a big leap forward in 3D light microscopic resolution and visualisation there was little hope to extend this to the world of ultrastructure. The only method available at the time to achieve real volume in an electron microscopy data set was serial ultra‐thin sectioning. One study from 1986 reconstructed 4 retinal amacrine cells from serial TEM sections^1^ and it took 4 years to complete, so for all practical purposes 3D correlative microscopy was still a pleasant but unachievable dream.

CLEM as a technique can be dated back to the1960s when pioneering microscopists such as Larry W. McDonald and Hans Dieter Geissinger attempted to use the resolving power of the electron microscope to add ultrastructural information to that produced by their light microscopic studies.^2^ These early CLEM studies were almost exclusively performed in two dimensions (2D) typically combining LM imaging of sliced tissues with either SEM or TEM. Beginning in the early 1980s the 3D information from these studies was increased through the use of confocal microscopes but adding 3D EM to CLEM studies was still very challenging.^3^ The obvious advantages of 2D CLEM were that the equipment was widely available, but the disadvantages were the lack of dimensional information resulting from the limited sample size in Z produced by SEM and TEM. TEM tomography could also produce results in 3D^4^; however, the volumes that could be studied were limited by the accelerating voltages of the TEMs used, and samples could normally not exceed a Z thickness of 300 nm. Thus, CLEM studies from the 1980s and early 90s lacked the depth of information that only comes with large correlative 3D volume electron microscopy (vEM) studies. Like all complicated techniques CLEM developed over time with many complex variations, some of which combined multiple advanced techniques such as colloidal gold labelling, live cell video microscopy, scanning electron microscopy (SEM) and high‐voltage transmission electron microscopy (hv‐TEM). While these studies remained rare, the numbers saw a small increase just after the year 2000 that became exponential in the next two decades. This increase parallels the growing increase in the establishment of institutional imaging cores where the expertise and equipment necessary for complicated workflows were available.

The increase in CLEM studies was also accelerated by a major breakthrough in 3D volume EM when in 1994 Richard J Young and colleagues published a study of the 3D ultrastructure of a mite, *Halarachnidae mesostigmata*,^5^ using a scanning electron microscope (SEM) equipped with a focused gallium ion beam that allowed fine milling of the specimen in the SEM chamber interspersed with serial imaging. The technology was termed focused ion beam scanning electron microscopy (FIB‐SEM) and had originated in the materials sciences as a method of nanofabrication. In 2006, Jurgen Heymann and colleagues expanded the scope of this technique to cells and tissues using the vEM data for 3D reconstructions of cellular membranes and organelles.^6^ Another technology was available for vEM of cells and tissues. In 1983, Steve Leighton and Alan Kuzirian working at the National Institutes of Health and the Woods Hole Marine Biology Laboratory developed a device for serial sectioning resin embedded blocks with a diamond knife placed within the SEM chamber.^7^ As personal computer technology and digital imaging were in the very early stages of development this complex instrument was never developed beyond the prototype stage. When computing technology had progressed the in chamber ultramicrotome was further developed by Winfried Denk and Heinz Horstmann for use in vEM of neuronal tissue.^8^ The process involved trimming and slicing to smoothen the face of a block of resin embedded tissue, imaging the blockface within the SEM, then slicing the blockface away to reveal a new tissue level and repeating the imaging process. This ‘slice and view’ technique was commercialised by Gatan and sold under the name of 3View and became known as serial block face scanning electron microscopy (SBF‐SEM). The advent of vEM technologies spurred on an increase in 3D correlative light and volume electron microscopy (vCLEM). Studies such as the ones by Bohumil Maco et al, Hannah Armer et al, and Lidia Llorca et al using FIB‐SEM,^9^, ^10^, ^11^ and those of Olivier Urwyler et al., and Robert Lees et al. using SBF‐SEM,^12^, ^13^. They employed high resolution 3D LM fluorescence for locating tagged proteins combined with 3D vEM to precisely match localisation information with ultrastructure. Other studies have combined vEM techniques to adapt the workflow to utilise the different advantages of the two technologies (Figure 1). Other techniques such as array tomography have also been used to increase the 3D information of EM studies and these techniques continue to be refined and developed.^14^

**FIGURE 1:**
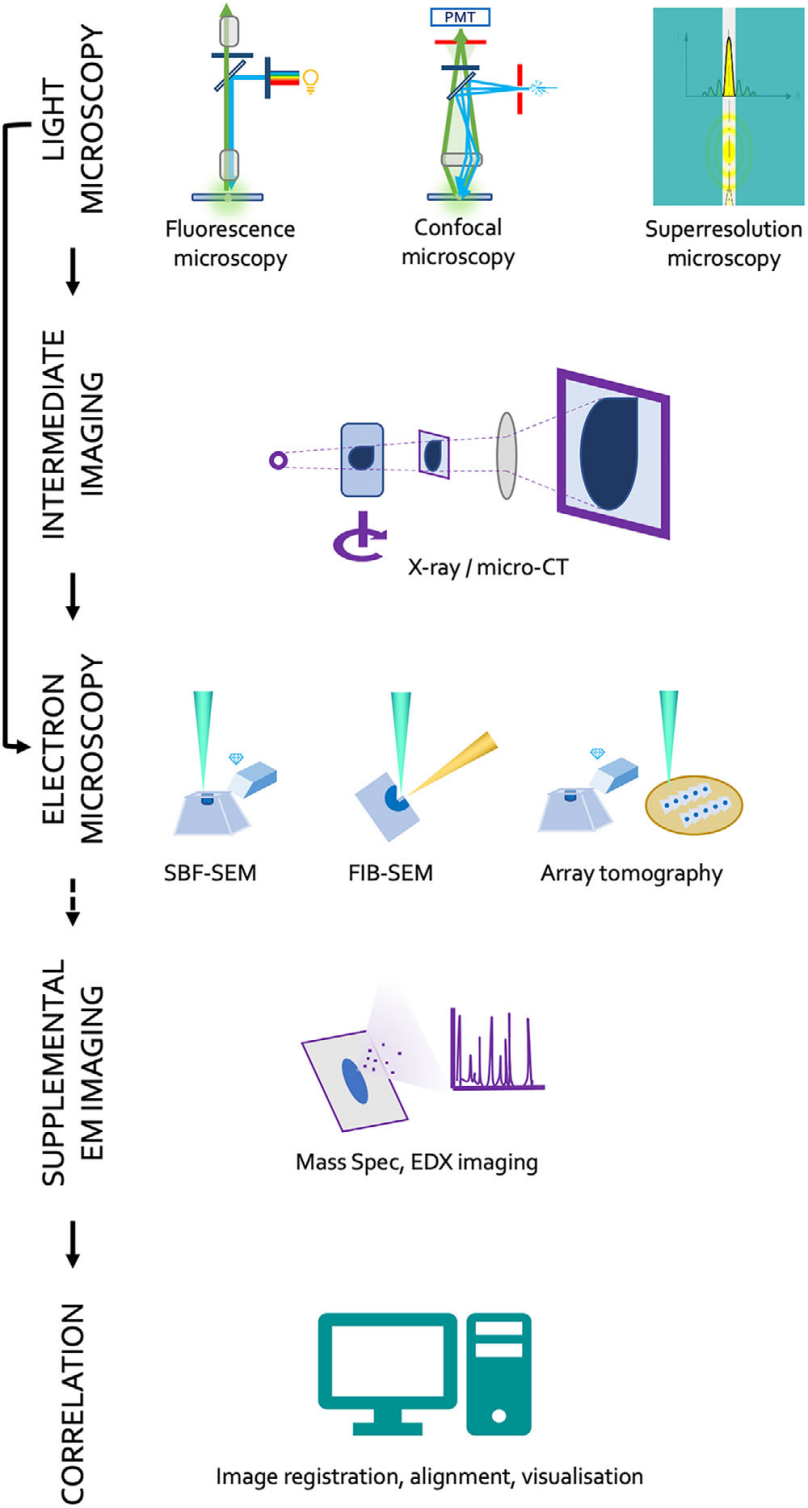
Possible workflows for vCLEM. A number of light microscopic imaging techniques can be incorporated into a vCLEM experiment with the aim of identifying particular structures of interest usually by fluorescently staining a protein or structure of interest. Intermediate imaging steps using X‐Ray microscopy or micro CT can further assist in identifying a region of interest (ROI) to be targeted in the electron microscope. Typical vEM methods used would be in chamber sectioning either with a diamond knife (SBF‐SEM) or a focused ion beam (FIB‐SEM) or external sectioning of a resin block followed by serial imaging (AT) of the resulting sections. Supplemental imaging techniques such as EDX or mass spectrometry can be used to provide elemental or protein signatures. Finally, the LM and vEM images and any associated data are compiled in the registered sections and visualisation is carried out via computer modelling of the data stack

While vCLEM studies are becoming more frequent the technologies and the techniques leading to successfully implementing these complex methods has taken some time to develop. In a large part this has been a community effort. In 2011, a meeting was held in Munich hosted by Carl Zeiss microscopy that brought together around 50 microscopists from Europe and the United States for 1 1/2 days of lectures and discussion concerning the future of CLEM as a 3D technique. Although at this time the techniques and workflows were in a very early stage of development, the enthusiasm for moving them forward was high. This small group formed the nucleus for a community centered around vEM and from that came the plan for a second larger and longer meeting that was held at the Vlaanderen Institute of Biotechnology (VIB) in Ghent in 2014. The organisers chose the title of ‘From 3D Light to 3D Electron Microscopy’ and a scientific committee was formed to construct the program and choose the speakers. Carl Zeiss provided financial and logistical support but left the specifics of the program to the scientific committee. The response from the scientific community to this new congress was enthusiastic, and the 160 available participant slots filled up quickly. In addition to the United States and Europe, participants came from Africa, the Middle East, South America and India indicating that the growing interest in vEM was international. The idea was to create a very interactive meeting that would combine lectures and discussion groups and there was a strong focus placed on practical workshops to demonstrate the instrumentation and techniques used in the vCLEM workflow. Workshops covered sample preparation, SBF‐SEM, FIB‐SEM and image processing. The meeting was highly successful and at the end, plans were put in place for another congress. Feedback from attendees indicated that a biennial schedule was preferred so it was decided to hold next congress in 2016 at the European Molecular Biology Laboratory (EMBL) in Heidelberg. Scientific sessions in this 3rd edition covered examples of CLEM methods, sample diversity, image processing and analysis and extending vCLEM by including other techniques such as energy dispersive spectroscopy (EDS),^15^ Micro CT^16^ and X‐Ray microscopy.^17^ Once again the registration slots were taken up rapidly and the program for this meeting very closely followed the 2014 Ghent format, interspersing lectures, workshops and discussion groups. Up until this point, the congress had focused on FIB‐SEM and SBF‐SEM but at this edition it was decided to add array tomography as it was a technique that could also provide 3D volume information and was becoming mature enough to attract a considerable number of laboratories and imaging core facilities to adopt it.^18^

The 4th edition was held in 2018, again in Ghent, and by this time it was obvious that many laboratories and imaging cores were adopting vCLEM techniques with the numbers of international participants and institutions represented increasing. Plans were made for a 5th edition of the congress to be held at the Francis Crick Institute in London in March of 2020. However, the SARS‐CoV‐2 pandemic forced its postponement. It was decided to hold the event online as a virtual congress and that took place in February of 2021. While the committee had some regrets that the warm and very personal nature of the preceding congresses would be lost it was more than made up for by being able to open the registration without limits thus making this edition more inclusive than before. In the end over 1450 participants registered representing more than 40 countries on 6 continents. It was challenging to adapt the format of the previous meetings to a virtual space, but again with support from Carl Zeiss the meeting was able to provide webinar based scientific sessions, interactive practical workshops and Zoom meeting format round table discussions. The meeting was held in 3 half‐day sessions to try and accommodate the many time zones of the participants and each day concluded with virtual open lounges in which participants could interact with speakers, workshop leaders and round table moderators.

One of the hallmarks of this event has been the very interactive nature of the congresses and the remarkable openness and level of networking and sharing that occurred. This has helped drive the vCLEM technologies forward and brought about many collaborations that have resulted in peer reviewed published works. When looking back on the different congress editions, one can notice an evolution in the discussions, which represents how vCLEM had evolved in merely a handful of years. The earlier editions covered practical details on getting started in vCLEM, explored which data reconstruction tools were available, and looked at possibilities to expand protocols and workflows to various sample types. The latest editions have focused on future challenges such as setting standards for data repositories and automating workflows. The congresses were an important barometer for ‘sensing’ and capturing the areas in which work needed to be done, and out of those interactions, a great many new tools and even some instrumentation advances have been developed. For example, discussions on the problems of sample charging led directly to the development of a focal charge compensation system in Mark Ellisman's laboratory at the National Center for Microscopy and Imaging Research that was later adopted and commercialised by Carl Zeiss.^19^ Other developments have been seen in the area of image reconstruction and analysis. The Microscopy Image Browser was significantly influenced based upon community discussions.^20^

As before, the 2021 congress was able to bring more new techniques and innovations into focus. In her Keynote lecture on array tomographic approaches Professor Kristina Micheva of Stanford University showed how the technique of Thomas Templier (MagC) uses a resin block containing magnetic particles as well as fluorescent beads to greatly simplify both serial section manipulation as well as alignment for reconstruction.^21^ The number of innovative correlative workflows also demonstrated how multi‐modal aspects of vCLEM are progressing, and examples combining novel approaches were presented including: XRay microscopy, Micro‐CT, elemental analysis using EDX and micro image mass spectrometry (CLEIMiT). In his keynote, Professor Wah Chiu of Stanford University showed that whole cell high‐voltage TEM cryo‐tomography could be used for vCLEM incorporating structural biology approaches to localise and examine individual protein molecules in situ.^22^ Image analysis and reconstruction, which has been a real bottleneck to widespread implementation of vCLEM, has also seen new developments based upon discussions in previous congresses. Perrine Paul‐Gilloteaux from Centre National de la Recherche Scientifique presented EC‐CLEM software for combining multi‐scale multi‐modal images,^23^ John Bogovic from Howard Huges Medical Institute demonstrated a deep learning based pipeline for image recognition and segmentation,^24^ Joris Roels of the Vlaanderen Institute of Biotechnology demonstrated an automated segmentation method using advanced transfer learning techniques^25^ and Ilya Belevich from the University of Helsinki talked about improvements to the microscopy image browser software using deep learning to automate the analysis of multiple datasets (DeepMIB).^26^ Although applying vCLEM on diverse biological specimens has been a topic of interest since the first edition, the 2021 conference covered a broad range of biological studies showing that the neuroscience focus of early vCLEM research had been widely extended. Another feature was the showcasing of many biological conclusions that could only be achieved through correlative volume microscopy. vCLEM has matured to a method that can push science forward by deepening our understanding of structure‐function relationships. The potential of correlating vEM with single cell data was highlighted in the closing Keynote by Yannick Schwab, who showed how this approach was used to create an atlas of gene expression profiles and cell morphology for the whole polychaete worm *Platinereis dumerilii*.^27^

In the virtual workshop sessions, practical tools and techniques were demonstrated and workflows for Confocal‐X‐Ray‐SBF‐SEM, array tomography, and targeted FIB‐SEM were presented.^28^ Carl Zeiss demonstrated new hardware to facilitate cryo‐vCLEM enabling a complete cryo‐workflow from widefield to confocal to FIB‐SEM. The workshops were hosted in multiple locations demonstrating that it is possible to use vCLEM techniques even if all the equipment necessary for a given workflow is not present in the same facility. They also presented possibilities for individuals without local access to high‐end vCLEM equipment to gain access through collaborations and initiatives such as Euro‐BioImaging.

As in previous years, the discussion of vCLEM issues by the community was indicative of both current challenges and long‐term goals. Besides troubleshooting and improving different steps in workflows, the community is also focusing on how to disseminate workflows and provide general access to technology and data. One of the round table discussions evolved around the need to ‘simplify’ workflows and how to set these up with ‘low tech’ solutions. Also the concept of executing a workflow across labs and facilities in different locations, an especially important strategy in times where people cannot travel to centres with specialised equipment, was discussed as a solution to provide access to technology. Proof‐of‐concept for this was highlighted in one of the workshops where EMBL and VIB performed a vCLEM experiment with samples and data (but not people) travelling between the two institutes. On the data front, there is a general consensus that sharing raw data is of utmost value, and although everyone agrees on the general philosophy a framework with ground rules and standards would be required to bring this idea into practice. On that subject comparison to other fields (like astronomy and protein databases) are looked to as models for best practice standards and practical implementation of tools for vCLEM repositories.

It is reasonable to ask why this technique and this niche area of EM has been so rapidly advanced by these congresses. Clearly, any meeting where like‐minded scientists gather will engender changes that advance science. However, to we who have attended, these meetings seem to have accomplished more in a shorter time. It could be that this small group of techniques, that appeared to be so challenging just 10 years ago, have evolved rapidly because of the relatively small group of scientists who use them needed each other's help to be successful. It is also true that these techniques require equipment that until recently was primarily found in imaging cores, and that two of these cores which have hosted congresses (VIB and EMBL) have as part of their mission educating the scientific community regarding the application of novel techniques. When the congresses began, there were no organised courses where vCLEM could be learned or textbooks that could provide guidance so that the congresses also acted as mini‐training courses. It is also true that these meetings have attracted a very stable group of participants, many from core imaging facilities whose willingness to share was not as hampered by individual competition, since serving their users needs was their principal goal. In any event it is clear that the rapidly increasing number of vCLEM publications has paralleled the initiation of the congress series and that many of these papers have come from people who have also attended the congresses.

vCLEM remains a developing technique, but it has proven it's potential to reveal novel structural information that was previously unappreciated or misinterpreted in 2D studies.^29^ What began as a rather far‐fetched idea grew into an achievable technique and is now moving into the mainstream of CLEM based research. A great deal of the credit should accrue to the microscopists who demonstrated the practicality and possibilities of this technology by working together and publishing their research. Significant credit is also due to companies such as Carl Zeiss and Gatan who developed the instrumentation and promoted the technique despite the fact that the global market was, at least at the beginning, quite limited. Out of the congresses, a vEM community has arisen and is actively involved in standardising, developing and promoting many aspects of vEM. The vEM community initiative is being coordinated by numerous scientists (https://www.volumeem.org/) and working groups are addressing sample preparation, training, data handling and analysis, outreach and instrumentation. Developing complex imaging techniques requires more resources than a single institution, company or individual can provide. Specialist scientific congresses, such as ‘From 3D Light to 3D Electron Microscopy’, are essential if disruptive technologies are to thrive and grow. The next edition, which is hoped to be a hybrid in person and virtual event, will be held at EMBL in Heidelberg March 13–16, 2022. While imaging across wide resolution scales in three dimensions once seemed a wonderful but impractical idea, the rise of vCLEM shows what can be accomplished by a few intrepid microscopists who believe in pushing boundaries to enable new ways of seeing the micro world.
